# Physicians’ and Patients’ Expectations From Digital Agents for Consultations: Interview Study Among Physicians and Patients

**DOI:** 10.2196/49647

**Published:** 2024-03-18

**Authors:** Andri Färber, Christiane Schwabe, Philipp H Stalder, Mateusz Dolata, Gerhard Schwabe

**Affiliations:** 1 ZHAW School of Management and Law Zurich University of Applied Sciences Winterthur Switzerland; 2 Department of Informatics University of Zurich Zurich Switzerland

**Keywords:** adherence to treatment, digital agents, eHealth, electronic medical records, health literacy, mobile health, mHealth, mobile phone

## Abstract

**Background:**

Physicians are currently overwhelmed by administrative tasks and spend very little time in consultations with patients, which hampers health literacy, shared decision-making, and treatment adherence.

**Objective:**

This study aims to examine whether digital agents constructed using fast-evolving generative artificial intelligence, such as ChatGPT, have the potential to improve consultations, adherence to treatment, and health literacy. We interviewed patients and physicians to obtain their opinions about 3 digital agents—a silent digital expert, a communicative digital expert, and a digital companion (DC).

**Methods:**

We conducted in-depth interviews with 25 patients and 22 physicians from a purposeful sample, with the patients having a wide age range and coming from different educational backgrounds and the physicians having different medical specialties. Transcripts of the interviews were deductively coded using MAXQDA (VERBI Software GmbH) and then summarized according to code and interview before being clustered for interpretation.

**Results:**

Statements from patients and physicians were categorized according to three consultation phases: (1) silent and communicative digital experts that are part of the consultation, (2) digital experts that hand over to a DC, and (3) DCs that support patients in the period between consultations. Overall, patients and physicians were open to these forms of digital support but had reservations about all 3 agents.

**Conclusions:**

Ultimately, we derived 9 requirements for designing digital agents to support consultations, treatment adherence, and health literacy based on the literature and our qualitative findings.

## Introduction

### Motivation

Consultations are less productive than what physicians and patients would wish [1,2], which hampers health literacy, shared decision-making, and treatment adherence. The recent rise of generative artificial intelligence (AI), such as ChatGPT, has sparked the interest of digital health developers, as they explore how this technology can improve shared decision-making, physician-patient communication, adherence to treatment, and health literacy. In this study, we sought to discover what physicians and patients expect from digital agents (functional requirements) and how this functionality should be provided (nonfunctional requirements). A user-centric perspective is essential for guiding the development of digital agents because it prepares physicians for changes in their consultation methods and allows patients to understand what the new technology can offer.

Through in-depth interviews (refer to the Methods section), we described 3 digital agents to physicians and patients, analyzed their impressions and expectations (refer to the Results section), and deduced a set of design requirements (refer to the Discussion section). An introduction to the related work and concepts for the 3 different digital agents is provided in the following sections.

### Related Work and Concepts

#### Relevant Medical Concepts

Overall, four medical concepts are essential when supporting medical consultations with digital agents: (1) shared decision-making, (2) physician-patient communication, (3) adherence to treatment, and (4) health literacy.

Consultations involve a participatory process between patients and physicians to reach an agreement regarding treatment goals and their implementation [3,4]. “Shared decision-making” has emerged as the gold standard for this participatory process [5-10] as it strives to reach a mutual agreement about therapy [6,7]. However, a systematic review of shared decision-making regarding clinical decisions found that the humanistic aspects of physician-patient communication were rarely assessed [11]. Good “physician-patient communication” is not only about technique or process but also involves understanding the whole person, finding common ground, and enhancing the patient-physician relationship [4]. In this way, physician-patient communication can have a therapeutic effect and influence health benefits [12].

The therapeutic process continues after the patient has left the consultation [3]. Once at home, it is up to the patient to implement the therapy plan, and the extent to which this occurs is referred to as “adherence to treatment” [13]. Adherence focuses on patients taking responsibility for their treatment and physicians collaborating more with their patients [14,15]. However, despite some progress, adherence to treatment remains insufficient [13,16-18]. First, there is a lack of “health literacy” when following the given instructions. Physicians may explain medical issues and treatment options during consultations, but their time is limited, and they must convey as much information as possible. Second, patients are in a stressful situation, which restricts their ability to absorb and hinders their recall [19-24]. Third, physicians may use medical terminology [25] with the following consequences: patients either do not understand or quickly forget what was discussed [26,27]. Brochures and leaflets are typically used to support health literacy, and modern approaches include video, multimedia, computer-assisted learning, mobile apps, and other web-based aids [28-32].

#### Digital Agents

Digital agents are computers that undertake tasks previously performed by humans. As such, they function autonomously, react to environmental situations, initiate actions, communicate with humans or machines, and behave intelligently [33]. An increasing volume of digitized data, improved algorithms, and better hardware has vastly enhanced the range of tasks that digital agents can perform. The most noticeable aspect is the recent success of generative AI. Nevertheless, the expanding capabilities of digital agents also raise concerns about AI in general and digital agents in particular [34]. Examples include their potential misuse, how they can be controlled, and whether they exhibit bias [35]. Besides these general concerns, researchers are interested in understanding exactly how digital agents interact with humans. Although humanlike behavior may be helpful in some situations, task performance may be impeded by excessive humanness [36,37] such as in situations where humans prefer a digital agent with a background function. This issue is critical in institutional settings [38], where professionalism is vital.

Discussion about the capabilities of digital agents and their suitability has also reached the medical domain [33,39,40]. Conceptually, the dyadic physician-patient consultation becomes triadic [41-44] if a digital agent is included. The presence of digital agents changes the consultation dynamics [45,46] and alters how patients and physicians behave [41]. Despite such insights, the discussion lacks a clear conceptualization of the digital agent’s role in the professional context of physician-patient consultation. Consequently, discussing what physicians and patients expect from digital agents during and between consultations has not been possible.

#### Current Digital Support for Consultation, Adherence to Treatment, and Health Literacy

Physicians use electronic medical records (EMRs) and encounter patient decision aids (PDAs) during consultations, which provides patients access to their data through patient portals. Patients may also store data in their personal health records (PHRs) and take advantage of mobile health (mHealth) apps between sessions.

EMRs support physicians in documenting medical history, including physical examinations and laboratory results. They are intended to reduce costs, improve patient safety, increase efficiency [47], and safeguard data [48,49]. As EMRs are designed primarily for documentation purposes [50], it is the physician’s responsibility to determine how to use them in patient interactions. Proper use of EMRs by trained health care professionals can improve health literacy and adherence to treatment compared with paper-based records [51], for example, if physicians share their EMR screens with patients during consultations [52,53]. However, when used ineptly, physicians lose control of the consultation owing to increased gaze shifts and multitasking, which hinders their medical reasoning [47,54]. In the presence of a computer, preexisting positive and negative communication skills are amplified [55,56].

Encounter PDAs support physician-patient consultations by providing decision-related information and choices [57-61]. Although they tend to be simple in design [61], physicians complain that lack of training and experience and insufficient content and format impede meaningful use of encounter PDAs [57,58]. Another challenge is keeping encounter PDAs updated with the latest information [60].

Patient portals provide patients with access to their data stored in EMRs [62]. In such tethered patient portals, the responsibility for maintaining the data lies with the physician. To be understood by patients, information from EMRs must be translated [62], and this applies to language, graphs, and other multimedia material.

Unlike patient portals, in electronic PHRs*,* patients themselves enter and maintain their health data [63]. Although PHRs can accumulate more information than patient portals, quality control and manageability are challenging. There is a consensus that more needs to be done (eg, patients also need to understand what they get from the PHR and need to act on what they understand) to enhance health outcomes or treatment adherence than just providing patients with access to their data [64,65]. Better-informed patients are not necessarily healthier patients [64], but there is (1) value and (2) potential in patient portals and PHRs. First, patients want access to their data to review it again at home, discuss it with their families, and use it as a starting point for further online research [62,64]. Second, there is evidence suggesting that patient portals and PHRs are more effective when they are interactive, when they are combined with other services such as reminders or interactive decision support, and when physicians actively promote their use [62,64].

Digital interventions based on mHealth apps promise to support patients’ health literacy and adherence to treatment. In 2017, >300,000 health apps were available in online app stores [66]. Not all are considered effective, convenient, or of high quality [67-69], and many have low success rates and high dropout rates [70-72]. Nevertheless, despite their limitations, mHealth apps appear to support patients effectively in treatment adherence [67,73,74]. If they pass the medical quality requirements, they can even be prescribed in the same manner as medicine [75,76]. Physicians are best placed to assist with their use, but this requires their integration into workflows and EMRs [74,77,78], and the security of patient data must be guaranteed [79].

#### Digital Agents to Support Consultation, Adherence to Treatment, and Health Literacy

##### Overview

We conceptualized 3 general roles for digital health agents, which tie together the modern medical concepts and previous studies of digital agents with current digital support for consultation, adherence to treatment, and health literacy. These served as a basis for our empirical study, when introducing our selected physicians and patients to digital agents.

A digital agent can be a “digital expert” that provides the right aids at the right time or offers a second opinion about diagnosis and treatment. It can stay in the background of the consultation as a “silent digital expert” or actively participate in the consultation as a “communicative digital expert.” Alternatively, it can be a “digital companion” (DC), which supports the patient between consultations. DCs provide patients with comprehensible information about diagnosis and ongoing treatment.

##### Silent Digital Expert

This is an extension of EMRs, providing the physician with contextual and real-time advice and additional information. The silent digital expert is designed to free the physician from searching vast information sources and allows more time for face-to-face consultation, thereby improving physician-patient communication [4,12]. For example, the silent digital agent can alert physicians to different diagnoses and drug interactions or offer prompts for further questions. The silent digital agent also supports diagnosis and suggests appropriate treatment in a shared decision-making process [5-10]. It acts as an aid to the physician and is visible and accessible only to the physician, and with patient consent, it can record, transcribe, analyze, and summarize the consultation.

##### Communicative Digital Expert

As the third party in a triadic consultation, the communicative digital expert offers the same functionality as the silent digital expert. However, it actively participates in the consultation by extending the functionality of EMRs and encounter PDAs through an agency. It may be physically represented as a humanlike robot, smart speaker, or device of any shape. As the third party, the communicative digital expert can be invited to comment about the decision-making process of physicians or patients [5-10] and become active in explaining medical topics, thereby improving health literacy [80-83]. As such, it can be considered as a physician’s assistant or patient’s advocate, thus improving physician-patient communication [4,12]. For example, it might interrupt the dialogue if a physician is very brief or dominant, thereby providing both parties with further information, diagnosis considerations, and treatment recommendations. It acts in an empathetic, patient-centered manner and is capable of identifying and taking patient preferences into consideration.

##### Digital Companion

This agent is intended to support patients *between* consultations by extending patient portals and PHRs and combining them with an mHealth app. It relies on data from EMRs and supports patient treatment behavior. Its primary goals are to improve the recall of recommendations and information, promote health literacy [80-83], and support treatment adherence [12-18,84]. DC captures the critical points of the physician-patient consultation, translates them into everyday language, enriches them with multimedia elements (audio, picture, diagram, and video), and makes them conveniently accessible to patients or their families at any time. It also provides the patient with curated additional information and interactively supports their health care education based on individual preferences. Using sensor data from various devices (eg, smartphones, smartwatches, pedometers, and blood glucose monitors) and patient’s interaction with DC, adherence to the treatment plan is measured, analyzed, and fed back to the patient (and with the patient’s consent, to the physician). DC provides context-specific, adaptive interventions [85-88] based on adherence measurement, individual treatment agreement, and patient preferences. For example, adherence support might include diet recipes, exercise instructions, morale-boosting talks, and so on.

## Methods

### Research Approach

This study aims to understand what physicians and patients require from digital agents. These requirements should be grounded not only on technical vision but also on current consultation practices, with a focus on problem-solving.

Our research approach was inspired by the practice-oriented approach popular in computer-supported cooperative work (CSCW). CSCW is an interdisciplinary field of research involving, among others, computer science, psychology, and sociology, to analyze the potential and the shortcomings of digital assistance in consultations [89-91]. CSCW mainly uses qualitative methods and focuses on how human collaboration can be supported by technical means [89,92]. As these means must be applied within a professional context, this also involves studying work practices from the perspective of those involved [93,94].

Our study embraced this tradition by following an exploratory paradigm, striving for deep, contextualized insights [95,96]. We conducted an interview-based qualitative study with 47 participants—22 (47%) physicians and 25 (53%) patients. Our analysis combined bottom-up thematic analysis and interpretive research, allowing for both broad coverage and deep insight.

Overall, the chosen methodological approach respected the need to understand patients’ and physicians’ perspectives regarding their work practices and the potential use of technologies. We addressed variation and triangulation, whereby multiple researchers conducted the interviews with different patients and physicians. We ensured audit throughout the process by mutual control among researchers and by assigning a quality manager role to one of the authors. The first author was directly engaged in data collection during a preliminary study [97] and guided data collection during this study to ensure adequate engagement in data collection activities. In summary, the study used various strategies to ensure the reliability and validity of the presented results [98] and followed the COREQ (Consolidated Criteria for Reporting Qualitative Research) guidelines for reporting qualitative research [99].

### Ethical Considerations

The Ethics Committee of the Zurich canton confirmed that this study was not subject to the Swiss Human Research Act (Business Administration System for Ethics Committees [BASEC]–Nr Req-2018-00847). Nevertheless, written informed consent was obtained from all participants before their interviews according to the World Medical Association Declaration of Helsinki [100].

### Sampling and Recruitment

Exploratory studies require a variety of opinions, but they do not seek to be representative. To ensure variety, we interviewed both physicians and patients. We also relied on purposive sampling using a maximum variation strategy [101], which allowed us to search for a broad range of physicians and patients. Given that 5 interviewers acquired the patients and physicians independently, we can assume the coverage to be better than that of strategies involving sampling through a single researcher. Table 1 shows the demographic characteristics of the study participants.

**Table 1:** Demographic data of the interviewed physicians and patients.

Characteristics			Physicians (n=22)		Patients (n=25)
**Sex, n (%)**					
	Male	12 (55)		14 (56)	
	Female	10 (45)		11 (44)	
Age (y), mean (SD; range)			50 (14; 25-66)		46 (19; 20-86)

Of the 22 physicians, 13 (59%) are active in primary care, and the others work in hospitals; 11 (50%) are general practitioners or specialize in internal medicine. Other specializations include pediatrics, gynecology, radio-oncology, and dentistry. The educational background of the 25 patients ranged from unskilled workers to professionals and academics. The patients presented a broad spectrum of conditions, including diabetes, multiple sclerosis, heart conditions, tick-borne encephalitis, and epilepsy.

We conducted 46 in-depth interviews that resulted in audio recordings with 32 hours of interview time, amounting to an average length of 42 minutes and 46 seconds (SD 13 min and 47 s). Of the 46 interviews, 45 (98%) were conducted with 1 interviewee per session, and 1 (2%) involved 2 respondents. The sample size assured data saturation—the topics emerging in the interviews began to overlap after about 18 to 20 interviews for each group [102]. Consistent with the practice for purposive sampling and maximum variation [101], we used various channels to establish the initial contact with the interviewees (email, face-to-face, and telephone). After confirming the time and date for a potential interview and giving their consent, no one dropped out of the study.

### Data Collection

In total, 5 researchers conducted in-depth interviews based on the respective interview guides—separate guides for patients and physicians [96]. The interview guides were developed based on the literature about physician-patient communication; adherence to treatment; existing solutions in the field of medical informatics; and the authors’ own experiences in the medical domain, including their research background. The overall structure of the interviews was informed by CSCW practice-oriented studies [93,94]. The interview guides were pretested in a preliminary study (with 11 health care professionals and 7 patients) published elsewhere [97]. Interviews for this study were conducted between January 2019 and May 2019, with patient interviews being conducted mostly in their homes and health care professional interviews in their professional setting. Before the interviews, all researchers underwent interview training sessions to ensure that they had the same understanding of the questions and knew how to conduct the interview. The interviews were structured around 3 areas: current situation or practice (format of and preparation for a consultation), future developments (expectations from and attitudes toward digital health care), and closure (other points that were not already covered).

When discussing about digital developments, we suggested potential ideas because users often lack the necessary imagination when asked about future products or services [103]. Nevertheless, when prompted, many users can express helpful, subjective opinions about specific ideas [103]. Therefore, in the spirit of design thinking [104], we exposed the users to key design ideas by describing the digital experts and DC and asking for their perceptions, expectations, and preferences regarding digital agent support. As is typical in design thinking, the discussion focused on the desirability of critical capabilities but did not include a detailed discussion about feasibility.

### Data Analysis

All the interviews were audio recorded and transcribed. The analysis combined deductive thematic research and interpretive research, allowing for broad coverage and deep insight simultaneously. During the top-down analysis, the transcripts were coded according to a codebook derived inductively from a small preliminary study [97]. A professor of nursing science cross-checked the codebook. Again, all researchers attended a training session to ensure that they had the same understanding of the codebook. All interviews were then deductively coded using MAXQDA (VERBI Software GmbH) [105]. The designated quality manager conducted quality assurance activities by controlling all code assignments and correcting them to ensure a consistent basis for analysis. We achieved thematic saturation—all themes from the specified coding schema appeared in the data with high frequency (the most frequent code was assigned 274 times and the least frequent was assigned 25 times; overall, we had 1954 assignments across all codes) [102]. Finally, all interviews were summarized by code; for each theme, we obtained a summary of participant opinions related to the code. These summaries formed the basis for further analysis, and the results were then used for interpretation.

To interpret the data, we organized 2 interpretation workshops involving the authors. The workshops aimed to establish a shared and consistent understanding of the most essential insights between the authors. The interpretive process involved iterative restructuring of the summaries along various dimensions, with 2 dimensions emerging as crucial for forming a consistent data view. First, we differentiated the problems, current practices that emerged to mitigate those problems, and potential technological solutions to address the problems that occurred during the interviews. Second, we observed that the issues aligned with the phases of a patient’s journey: (1) consultation, (2) “transition” between consultations and period between consultations, and (3) actual period between consultations. These differentiations provided the framework for reporting our results, and the proposed structure covered all the challenges and problems identified during coding.

In our presentation of the results, we refer to the frequency of specific challenges because, after identifying the framework and distributing the significant challenges for each element in the framework, we returned to the coded data to classify the coded passages. In the following section, we have presented the quantified data about the frequency of passages pertaining to the challenges. However, it is important to clarify that we do not assert the representativeness of these figures, as the analyzed population was not chosen to be representative of the broader population. Instead, the numbers ensured the thematic saturation mentioned previously.

## Results

Through analysis, we categorized the results into 3 steps in the patient journey: first, the consultation; then, incorporating information from the consultation into their lives; and finally, the time between consultations.

### Problems and Agent-Based Solutions During a Consultation

During consultation, the main challenge, according to physicians and patients, is conveying complex information in minimal time to laypeople with various backgrounds, expectations, and abilities while building or maintaining a relationship of trust. Table 2 summarizes the problems voiced by physicians and patients, current practices (as presented by the interview partners), and envisioned solutions offered by the 2 different versions of digital experts.

**Table 2:** Problems and solutions suggested during a consultation, along with the number of mentions in interviews.

Problems during the consultation	Current practices	Solutions offered by the silent digital experts	Solutions offered by communicative digital experts
Time pressures (physicians: 5/22, 23%; patients: 3/25, 12%)	—^a^	Physicians can concentrate on a thorough and engaging consultation using digital situational information	Physicians can concentrate on a thorough and engaging consultation using digital situational information
Medical information is complex (physicians: 9/22, 41%; patients: 18/25, 72%)	Physicians use graphics, visualizations, videos, and 3D models from brochures, books, and online sources (physicians: 14/22, 64%; patients: 7/25, 28%) Physicians draw illustrations themselves (physicians: 13/22, 59%; patients: 4/25, 16%)	The digital expert provides physicians with the following: The right visual aid at the right time Graphic templates or blank drawing areas that they can use for their own drawings	The digital expert suggests text, images, audios, and videos tailored to individual patient needs
Not all patients respond to medical advice and information in the same manner (physicians: 12/22, 55%; patients: no matching question)	Most physicians try to approach patients individually by adapting their language to a patient’s educational background or medical knowledge (physicians: 4/22, 18%; patients: no matching question)	The digital expert provides visual aids tailored to a patient’s educational background, numerical ability, or language skills	The digital expert intervenes if it determines (eg, through sentiment analysis [106]) that a patient does not understand the physician
Patients expect more transparency and control over the treatment process (physicians: 2/22, 9%; patients: 21/25, 84%)	Many patients engage in conversations with physicians and take responsibility for their treatment (patient: 3/25, 12%), and physicians try to support this (physicians: 4/22, 18%; patients: 5/25, 20%)	—	The digital expert intervenes when physicians do not give their patients enough time to talk, and it can empower patients to take more control
Some patients do not agree with the proposed treatment plan (physicians: 4/22, 18%; patients: 6/25, 24%)	Physicians respond with more intensive explanations (physicians: 8/22, 36%) Physicians protect themselves by documenting the conversation Physicians do not enforce treatment	The digital expert offers arguments, statistics, and figures to support the physician’s point of view	The digital expert advocates for the patient (by putting the physician’s thoughts or guidelines into perspective) or for the physician (by supporting the physician’s thoughts or guidelines)
The computer distracts the physician and interrupts communication, and use of computer amplifies inferior communication skills	—	The user interface of the digital expert is designed to be self-explanatory and user-friendly Instead of the physician, the digital expert searches for information and offers context-related content	The digital expert supports the physician and the patient, for example, through active listening It will only interfere by assisting an already impaired conversation

^a^Nothing mentioned in the interviews.

Regarding current practices, patients and physicians report that there is very little time for a thorough and engaging conversation:

I just felt like I was being processed. Quick assessment with the question: What’s the problem? And I felt that I couldn’t even say what I had because it was already clear to the physician. After a quarter of an hour, I was out of there again, and I was no wiser.

Male patient; aged 60 years; D07

I frequently make lifestyle recommendations. Costs time too, by the way, cannot be done in a 20-minute consultation that’s just long enough for issuing a prescription.

Male general practitioner; aged 64 years; hospital; ST09

Most physicians in this sample practice shared decision-making. Some use the explicit term during the interview, whereas others simply implement shared decision-making without labeling it as such:

Then I say, we could try pharmacy, we could try herbs, we could try acupuncture or this or that. I’ll let the patient have a say. Because then the patient’s adherence is also much better.

Female general practitioner; aged 65 years; medical office; MA10

All interviewed patients favored a silent digital expert as an aid to the physician; they did not object to physicians using online sources to obtain additional information during a consultation:

I don’t like having a doctor who introduces him- or herself as “I am the all-knowing one.” For me, that tends to inspire confidence when a physician says: I don’t know, I have to work with the exclusion procedure.

Male patient; aged 74 years; F01

However, patients expect uninterrupted attention, which requires a sufficiently high level of expertise by the physician in using the computer:

He kept asking and reading to me while he was writing and asking me if that was correct. This was great for me because then I knew what he was writing.

Female patient; aged 52 years; S10

Most physicians in this study would welcome a silent digital expert to facilitate multitasking, and some already use drug interaction assistants, risk or score calculators:

You can’t read through the books in the evening. That would mean an insane amount of time or such a head. That’s why these are important tools, I think for rare conditions it’s certainly a good idea.

Female gynecologist; aged 35 years; hospital; MA02

However, the benefits of a digital expert are assessed differently by those in different medical disciplines. A physician was concerned about the transfer of responsibility to the digital expert, whereas another physician worried about a decline in interprofessional communication. A young physician was concerned that this would cause them to acquire very little experience and self-confidence:

You rely too firmly on that afterward. Then you believe too firmly in that. Then it takes over your task, so to speak.

Female dentist; aged 29 years; dental surgery; MA03

Most patients in this sample view communicative digital experts positively. Those against them are concerned that they might be disruptive or could be manipulated by the physician:

I do not know what the physician can enter there, and then it is clear that the computer represents the opinion of the physician.

Female patient; aged 51 years; S07

The opinions of those in favor of it differ. Some consider a communicative digital expert as helping less skillful physicians and others consider it as helping competent physicians. Some would like a digital expert to be a physician’s assistant, whereas others consider it as a patient’s advocate:

As a patient, you are always subordinate to the physician, in that sense. I don’t think it’s a bad thing when someone else is on my side.

Female patient; aged 28 years; S06

Approximately two-thirds of the interviewed physicians reject the communicative digital expert. For them, credibility, decision-making authority, and their patients’ trust are at stake. Some consider empathy between the physician and patient as essential for patient adherence to treatment and, therefore, do not believe that a digital expert can help. A physician found communicative digital experts annoying but assumed that physicians and patients would get used to them over time:

In principle, I say, there is still an interpersonal level that artificial intelligence cannot comprehend.

Female general practitioner; aged 48 years; medical office; MA08

### Problems and Agent-Based Solutions for Transitioning From Consultations to the Period Between Consultations

Problems during the consultation may also hinder treatment because poor consultations can impair health literacy and adherence to treatment. Table 3 provides an overview of the voiced consultation issues that affect the time between consultations and the envisioned solutions offered through an interaction of the digital expert and DC.

**Table 3:** Problems and envisioned solutions for transitioning from consultations to the period between consultations, along with the number of mentions in the interviews.

Problems resulting from the consultation	Current practices	Solutions offered by the digital experts connecting to the digital companion
Patients cannot remember everything that the physician says (physicians: 0/22, 0%; patients: 10/25, 40%)	Patients do the following: Bring companions to the consultation Consult brochures or online sources (patients: 2/25, 8%) Use reminders on smartphones (patients: 2/25, 8%) Take notes (patients: 6/25, 24%) Physicians do the following: Repeat (physicians: 2/22, 9%) Use active listening techniques	The digital expert records, transcribes, and summarizes the conversation for the patient (quality assurance)
Identifying and introducing clinically relevant mHealth^a^ apps is time consuming and difficult	Patients search for apps themselves, but use dropout rates are high	The digital expert suggests quality-assured mHealth apps or equivalent features of the digital companion

^a^mHealth: mobile health.

Most physicians in this sample see potential in automated recording and transcription. A physician hoped that digital experts would give them more time to communicate with patients. However, physicians doubt whether a computer can separate relevant statements from irrelevant ones and produce relevant summaries. Some physicians stress that the notes they make for themselves about the case cannot be directly shared with the patient but need to be translated. Others insist on control over the information that is shared with patients:

Therefore, the software must either be able to guarantee this or otherwise it is legally difficult to prove that the patient has been informed correctly.

Male radio-oncologist; aged 35 years; hospital; MA01

Besides technical difficulties, the interviewed physicians see another reason to avoid automatic summaries—subjective perceptions are often only discussed verbally or communicated via telephone owing to fear of litigation:

Certain things, incidents and so on, or special experiences or special stories that are told that could have legal relevance. I don’t list them in the computer.

Male general practitioner; aged 62 years; medical office; ST02

Another physician takes precisely the opposite position. They would appreciate transcripts of complex consultations in which, for example, discussions about child protection or off-label prescriptions of medication are involved. A physician did not believe that a consultation’s significant first and last seconds would be transcribed with the necessary weighting.

Patients also have different opinions about digital experts. Only a few patients in this study raised data protection concerns regarding the consultation transcripts and other information recorded during the consultation. Some patients indicated that they would benefit from this evidence of what was said in the event of disagreement or malpractice. A patient was worried about a decline in care because physicians were afraid of malpractice lawsuits:

I tend to think I get worse treatment because most physicians have way too much fear of someone coming in afterward and saying, “I’m going to sue you – you told me something wrong.”

Male patient; aged 61 years; S02

### Problems and Agent-Based Solutions for the Period Between Consultations

The consultation cannot cover all the questions and issues arising between consultation appointments, and patients must rely on their own judgment or a tool that assists them during this period. Table 4 presents the problems that arise between consultations that lead to poor adherence and the solutions offered by DC.

**Table 4:** Problems and envisioned solutions for the period between consultations, along with the number of mentions in the interviews.

Problems arising between consultations	Current practices	Solutions offered by DC^a^
Patients lack information because of the following: Insufficient time for explanations during the consultation (physicians: 5/22, 23%; patients: 3/25, 12%) Poor recall of the consultation (physicians: 3/22, 14%; patients: 11/25, 44%) More questions arising later (physicians: 0/22, 0%; patients: 11/25, 44%)	Patients do the following: Use online sources, but they are skeptical, and some distrust online forums in particular (physicians: 6/22, 27%; patients: 6/25, 24%) Read brochures (patients: 3/25, 12%), attend public lectures, or even attend anatomy courses Physicians provide brochures to guide patients away from online self-diagnosis (physicians: 2/22, 9%).	DC provides curated content and web links tailored to the patient’s diagnosis. This reduces misinformation and false self-diagnosis. In addition, it fosters more trust in health care information.
Patients lack clear instructions and specific information but instead experience information overload (physicians: 0/22, 0%; patients: 8/25, 32%)	Physicians provide paper-based instructions regarding medication, exercises, and lifestyle changes (physicians: 3/22, 14%; patients: 8/25, 32%)	DC tailors content to patient preferences, contexts, and specific circumstances. This includes content presentation in different formats (simple or sophisticated text, images, audios, and videos).
Patients are on their own between consultations (physicians: 3/22, 14%; patients: 1/25, 4%)	Patients report little interaction with their physicians between consultations Some use email but only sparingly (physicians: 2/22, 9%; patients: 7/25, 28%)	DC provides low-barrier access to the physician between consultations. A chatbot covers part of the conversation to protect physicians from huge workload.
Patients are overchallenged when taking their medication (physicians: 2/22, 9%; patients: 8/25, 32%)	Medication apps can support complicated medication regimes (physicians: 1/22, 5%; patients: 2/25, 8%)	DC supports adherence by providing the patient with individualized interventions that consider patient preferences, contexts, and specific circumstances.
Treatment success or failure goes unnoticed (physicians: 4/22, 18%; patients: 0/25, 0%)	Physicians ask patients to maintain diaries or journals, mostly paper based (physicians: 8/22, 36%; patients: 3/25, 12%)	DC offers easy-to-maintain diaries and journals, including data captured from digital devices (eg, wearables). The collected data can be shared with physicians (with the patient’s consent).
Measuring adherence is difficult	Adherence is rarely measured, and often, it is only based on the purchase of medicines (physicians: 1/22, 5%; patients: no corresponding question)	DC offers adherence measurements in an easy-to-understand format.

^a^DC: digital companion.

Most patients in this study would welcome a DC; however, a few are skeptical or undecided. Patients are open to using electronic tools and online services regarding current practices. However, this is not always helpful to physicians:

People practically come with a diagnosis, and after that, we first have to come back to the symptoms. And I have to say, “hey, we have to start all over again.”

Male general practitioner; aged 66 years; medical office; ST01

Many physicians who were interviewed could see the potential of a DC. Some hoped this would improve adherence to medical advice, whereas a physician saw a significant benefit in making the DC genuinely personalized and tailored to an individual patient’s needs. Regarding monitoring patient behavior between consultations, less than one-third of the physicians reported adherence measurement (which is usually based on the purchase of medications):

That’s why I’m very happy when the patients order medication from us because then I can see on the computer when they have picked up their medication. I don’t see that when they buy medicines from the pharmacy.

Female general practitioner; aged 48 years; medical office; MA08

Most physicians in this sample are open to receiving and interpreting monitoring data from patients and their mobile devices. However, they have the following reservations. First, there is an unmanageable number of mobile apps. Second, they fear data overload and being forced to respond to monitoring results, which requires additional time that physicians do not have. Third, physicians see a risk that such monitoring will negatively influence patient behavior. A physician raised the possibility that neurosis could result from constant introspection. Another concern was that patients would abdicate responsibility for their condition by transmitting data and threshold violations. Despite these concerns, confronting patients regarding their threshold violations encourages them to reflect on their condition and possible lifestyle changes. Therefore, patients can become “experts” on their condition:

Because that is certainly one aspect when patients think about it: Why did my sugar do that now? That’s the most instructive. And the goal is that they become the “expert” and I coach them.

Female general practitioner; aged 39 years; hospital; ST08

## Discussion

### Overview

Problems in physician-patient interaction that ultimately hamper treatment adherence can be classified into 3 categories: problems regarding the consultation itself, problems from the consultation but appearing between consultations, and new problems arising between consultations. These problems overlap and, therefore, need to be addressed using integrated support systems. On the basis of the scenario, a support system consisting of digital agents assisting in the consultation and a companion for the periods between consultations is proposed. To qualify for the task, these agents need to meet the expectations of physicians and patients and improve health outcomes. In the following sections, we discuss design recommendations for the 3 digital agents that are active in the consultation and act as the patient’s companion between consultations.

### Requirements for Digital Experts During the Consultation

Digital experts reveal their capabilities during the consultation by integrating and extending the functionalities of EMRs and encounter PDAs with the characteristics of digital agents [33]. These include autonomous and intelligent behavior, reactions to environmental situations, and communication with humans or machines.

#### The Digital Agent Should Make Its Role in the Triadic Consultation Transparent

Our interviews asked for opinions about including medically skilled digital agents as part of a physician’s EMR [45,46]. These can facilitate conversations between physicians and patients or offer second opinions regarding diagnosis and treatment. In such cases, the digital agent functions as an additional physician. Although most patients would welcome this triadic consultation, some fear that physicians could manipulate their DCs. These reservations arise from an understanding that digital agents could adopt the role of a second physician and a trusted family member, spouse, or friend [41,42]. Such roles include informational or emotional support (eg, taking notes, ensuring understanding, and reassuring patients) [42]. Accordingly, the role of a digital agent in consultation must be clearly defined and transparent to patients. Further studies might explore what patients require to trust and benefit most from these digital agents in the role of a second physician, family member, spouse, or friend.

#### The Digital Agent Should Encourage Trust and Support the Physician-Patient Relationship While Safeguarding the Physician’s Credibility

The literature and interviews with physicians and patients agree on the importance of trust and good relationships between physicians and patients in a medical setting [4,12]. Although traditional health IT (eg, EMRs and encounter PDAs) does not seem to interfere with patient-physician relationships [53], the situation changes when digital agents act as medical experts or DCs during a consultation. Most interviewed patients like the idea of a digital agent and do not think it will harm the physician-patient relationship. At the same time, many physicians have an opposing view, fearing loss of credibility and decision-making authority. Therefore, a challenge for DC is to foster trust and support, rather than undermine, the relationship between physicians and patients. Such digital agents must support patients but not unduly contradict physicians or disrupt the natural flow of conversation. This means that digital agents must recognize whether a piece of medical advice will strengthen or damage the relationship.

#### The Digital Agent Should Help Physicians to Focus on the Patient During the Consultation

The interviewed patients expect their physicians’ full attention even when interacting with a computer. In a traditional practice setting, computer screens create a barrier between patients and physicians and can be a serious distraction [47,54]. However, digital agents act independently or are triggered by voice control to provide information or document the conversation, requiring less attention from the physician. The form of digital agents integrated into the conversation can range from shared screens or smart speakers to humanlike robots. Technological advances have brought such user interfaces and digital agents more close to reality. Further studies should indicate what patients and physicians are most likely to accept.

#### The Digital Agent Should Support Physicians by Taking Over Administrative Duties

Administrative duties prevents physicians from doing what they were trained to do (at considerable expense) and reduces their job satisfaction. The time pressure resulting from these administrative duties is a well-known problem that affects patient health outcomes [1,2,12]. This issue surfaced in the interviews with physicians and patients who were dissatisfied with their treatment. Therefore, a significant role for digital experts is to relieve physicians from as many administrative duties as possible. However, it is essential for physicians that their medical reasoning is considered as something more than mere administration. Recording, transcribing, and summarizing the conversation is necessary, but it is not the whole story. Digital experts should support medical reasoning of physicians and ask for it if not already done, rather than impeding it.

### Requirements for Handover From Digital Experts to DCs

To ensure a seamless patient experience, information collected and discussed during the consultation must be passed from the digital experts supporting the consultation to a patient’s DC.

#### The Digital Agent Should Tailor Information and Patient Education to Individual Patient Needs and Preferences

In supporting consultation, digital experts could, for example, provide appropriate information at the appropriate time. After consultation, DCs could continue patient education between consultations, which is tailored to their information needs and preferences. This can give physicians extra time during consultations [1,2] and assist patients in recalling recommendations and information [19,23,24]. In contrast to reading widely circulated brochures, leaflets, and generalized online sources [28,29,31,32,107], patients receive personalized information matching their specific circumstances and treatment plans. This saves time by reducing the need to guide patients away from potentially incorrect self-diagnosis [30].

Our interviews indicated that physicians effectively tailor information to their patients’ needs and backgrounds. Therefore, digital agents in the form of digital experts and companions must keep up with or even outperform physicians to add value. To achieve this, digital experts should either be able to draw on predefined patient profiles or interpret and assess patient preferences and backgrounds correctly. Physicians understandably insist on maintaining overall control as they are liable for the information they give their patients. A suboptimal solution would require physicians to verify the information they provide patients via the DC. In contrast, a better solution would ensure (in a trusted manner) that the information offered was consistent with the physician’s directions.

### Requirements for the DC in the Period Between Consultations

DCs support patients as digital agents between consultations by integrating and extending the functionalities of patient portals, PHRs, and mHealth apps.

#### The Digital Agent Should Offer Adaptive Interventions for Behavior Change

In conventional lifestyle change treatment, adaptive interventions are standard, and physicians and patients adapt and agree about the treatment every few weeks or months, ideally in a shared decision-making process [3,4,6,7,9]. However, adjustment cycles are dependent on consultation cycles, and in the meantime, patients may treat themselves incorrectly or discontinue a treatment owing to a lack of corrective measures. Here, digital agents in the form of DCs can shorten the cycle considerably. Depending on a patient’s mood, context, experience, and feedback, the DC can adjust the treatment within days, hours, minutes, or even seconds [85,86]. In our interviews, patients welcomed the idea of such functional flexibility. However, the challenge for the digital agent is to offer adaptive interventions that align with the respective physician’s recommendations, comply with medical device regulations, and fulfill safety and performance requirements. Further studies must demonstrate that this type of adaptive intervention will improve treatment adherence.

#### The Digital Agent Should Measure and Monitor Patients’ Adherence to Treatment and Provide Physicians With Easy-to-Read and Easy-to-Interpret Summaries

Measuring patients’ adherence to treatment is a prerequisite for adaptive interventions [13]. Our interviews indicate scope for improvement regarding the measurement of treatment adherence—particularly for exercise and lifestyle changes. DCs are well suited to measure adherence based on objective data from sensors and subjective data such as chatbot conversations with patients. The interviewed physicians indicated that they would accept patient behavior monitoring if DCs aggregated the monitoring results and communicated them directly to EMRs. The literature also calls for this type of workflow integration [62,74,77,78]. However, the DC must be able to recognize red-flag situations and respond appropriately because the responsibility and workload of constantly monitoring the results cannot solely rely on physicians.

Further studies are needed to determine how patients respond to behavioral monitoring. The interviewed physicians anticipate positive effects, such as patients becoming “experts” on their condition, and adverse effects, such as patients relinquishing responsibility for their actions. Therefore, digital agents must monitor patients in a supportive manner and report the results in a form that assists rather than overloads the physician.

#### The Digital Agent Should React to Feedback and Questions From Patients in the Period Between Consultations

The more sophisticated the DC’s communication and interaction skills are, the greater the expectation patients have for them to react appropriately. It is insufficient to simply give patients access to information through patient portals or PHRs [62,63] or have chatbots handling patient questions and feedback. In certain circumstances, patients still wish to talk to their human physician. In such cases, a triage mechanism might involve physicians only when necessary. However, the associated liability issues affecting the physicians (eg, in the case of suicidal intent) must be resolved.

### Requirements for the Integration of Digital Experts and DCs

Only the integration of digital experts and DCs can unlock the full potential of these agents to support the entire consultation process for the mutual benefit of patients and physicians.

#### The Digital Agent Should Integrate Consultation Support (Digital Experts) and Patient Apps (DCs)

Integrating digital experts and DCs closes the loop from one consultation to the next and synergistically increases the benefits of both agents [108]. From a digital expert to a DC, personalized information about the diagnosis and treatment is transmitted immediately at the end of the consultation. This avoids media discontinuity, overcomes the problem of poor recall of recommendations or information, and allows patients to implement correct therapy immediately. Some of this functionality is already part of patient portals or PHRs [62,63]. However, making this information available in an mHealth app supported by digital agents allows for better interactivity, adherence support, and measurement. As access to information alone has not proven to be effective [64,65], the mHealth approach promises greater effectiveness. Adherence measurements are fed from the DC to the digital expert based on sensor data and patient-reported outcome measures (eg, diary entries and chatbot threads). This allows physicians to prepare for the next consultation and saves time because patients do not have to report verbally what they have already entered into the app. The interviewed physicians and patients welcomed this focus and time-saving measure, and the literature also calls for workflow integration along these lines [77,109-111].

### Limitations

We derived the requirements for the design of digital agents to support consultation, adherence to treatment, and health literacy solely based on the statements obtained from our in-depth interviews with patients and physicians. Therefore, the 9 resulting requirements cannot be described as exhaustive. In particular, many necessary nonfunctional requirements are still lacking.

Furthermore, this study was conducted in Switzerland, which has one of the most expensive health care systems in the world. According to participating physicians, the standard consultation time is 20 minutes, which is significantly longer than that in many other countries. The responses from patients and physicians in other places and cultures might differ considerably. Further limitations may have arisen from the nature of a qualitative study based on a purposive sample. Although such a study results in a broad picture and deep insights, it may not be representative, not even for Switzerland. In addition, it is impossible to quantify the importance of the issues, suggested solutions, participant feedback, or the derived design requirements. For such purposes, surveys based on the insights obtained from this study are better suited. In addition, we cannot draw any conclusions related to specific user groups or medical disciplines. The fact that interview partners from very diverse backgrounds made similar observations and judgments indicates that our findings could be applied to various disciplines and user groups.

### Conclusions and Future Studies

With the introduction of generative AI such as ChatGPT, the time for digital agents to support consultation, adherence to treatment, and health literacy may have arrived. There is enormous potential for patients and physicians to benefit from this new technology. Through in-depth interviews, both parties revealed their opinions about a silent and a communicative digital expert to support consultation and a DC to accompany patients between consultations. Their responses are synthesized into the following 9 requirements for the design of digital agents to support consultations.

The digital agent should do the following:

1. Make its role in the triadic consultation transparent
2. Encourage trust and support the physician-patient relationship while safeguarding physician credibility
3. Help physicians to focus on the patient during the consultation
4. Support physicians by taking over administrative duties
5. Tailor information and patient education to individual patient needs and preferences
6. Offer adaptive interventions for behavior change
7. Measure and monitor patient adherence to treatment and provide physicians with easy-to-read and easy-to-interpret summaries
8. React to feedback and questions from patients in the period between consultations
9. Integrate consultation support (digital experts) and patient apps (DCs).

Some recommendations for future studies were also offered in Requirements for Digital Experts During the Consultation section and Requirements for the DC Between Consultations section in the Discussion section. In addition, we suggest the following:

1. Obtain a complete set of requirements for the design of digital agents for consultation; a full requirement engineering approach would need to be followed and explored in the field. This would include an analysis of the technical feasibility and economic viability [104] of the system, with the results of this study serving as a starting point.
2. Depending on where the digital agents are to be deployed, this study could be replicated with local patients and physicians.

## Abbreviations

AI: artificial intelligence
COREQ: Consolidated Criteria for Reporting Qualitative Research
CSCW: computer-supported cooperative work
DC: digital companion
EMR: electronic medical record
mHealth: mobile health
PDA: patient decision aid
PHR: personal health record
